# Combining EQ-5D-5L items into a level summary score: demonstrating feasibility using non-parametric item response theory using an international dataset

**DOI:** 10.1007/s11136-021-02922-1

**Published:** 2021-07-08

**Authors:** You-Shan Feng, Ruixuan Jiang, A. Simon Pickard, Thomas Kohlmann

**Affiliations:** 1 Institute for Clinical Epidemiology and Applied Biometrics, Medical University of Tübingen, Silcherstraße 5 72076, Tübingen, Germany; 2 Institute for Community Medicine, University of Greifswald, Greifswald, Germany; 3 Center for Observational and Real-World Evidence, Merck & Co, Kenilworth, NJ USA; 4 College of Pharmacy, University of Illinois At Chicago, Chicago, IL USA

**Keywords:** EQ-5D-5L, Non-economic scoring approaches, Non-parametric item response theory, Mokken scaling, Unweighted summary score

## Abstract

**Background:**

The EQ-5D-5L is a well-established health questionnaire that estimates health utilities by applying preference-based weights. Limited work has been done to examine alternative scoring approaches when utility weights are unavailable or inapplicable. We examined whether the Mokken scaling approach can elucidate 1) if the level summary score is appropriate for the EQ-5D-5L and 2) an interpretation of such a score.

**Methods:**

The R package “mokken” was used to assess monotonicity (scaling coefficients H, automated item selection procedure) and manifest invariant item ordering (MIIO: paired item response functions [IRF], H^T^). We used a rich dataset (the Multiple Instrument Comparison, MIC) which includes EQ-5D-5L data from six Western countries.

**Results:**

While all EQ-5D-5L items demonstrated monotonicity, the anxiety/depression (AD) item had weak scalability (H_i_ = 0.377). Without AD, scalability improved from H_s_ = 0.559 to H_s_ = 0.714. MIIO revealed that the 5 items can be ordered, and the ordering is moderately accurate in the MIC data (H^T^ = 0.463). Excluding AD, H^T^ improves to 0.743. Results were largely consistent across disease and country subgroups.

**Discussion:**

The 5 items of the EQ-5D-5L form a moderate to strong Mokken scale, enabling persons to be ordered using the level summary score. Item ordering suggests that the lower range of the score represents mainly problems with pain and anxiety/depression, the mid-range indicates additional problems with mobility and usual activities, and middle to higher range of scores reveals additional limitations with self-care. Scalability and item ordering are even stronger when the anxiety/depression item is not included in the scale.

**Supplementary Information:**

The online version contains supplementary material available at 10.1007/s11136-021-02922-1.

## Background

The EQ-5D is a widely used generic measure of health [1, 2]. As it is brief and not disease specific, the EQ-5D is applied in a broad range of settings, including measurement of health status in clinical practice, population health surveillance, assessment of healthcare quality, medical decision making, and patient communication [3–9]. The EQ-5D-5L expanded the response levels to five from the original three-level version (EQ-5D-3L) [10].

The EQ-5D is best known for the generation of quality-adjusted life years (QALY) in cost-utility analysis, used to inform drug reimbursement and pricing decisions in some countries/regions. Utility values, which are used to estimate QALYs, are calculated for EQ-5D-5L health states by applying a societal value set. Societal value sets are preference-based scoring weights estimated using valuation studies [11]. In valuation studies, hypothetical EQ-5D-5L health states are valued using choice-based methods, such as the time trade-off. These studies are generally conducted using representative, location/region-specific population samples. However, for many applications of the EQ-5D, population/country-specific utility scores may be unjustifiable or even introduce additional statistical biases [7, 9, 12]. An alternative method to summarize the instrument, relevant when utility weights are unavailable or unsuitable (e.g., EQ-5D-Y), is a total sum score of the severity levels on each dimension. Because each item of the EQ-5D has the same number of response levels, all items and severity levels contribute equally to this additive score. This approach has been termed “equally weighted” score [13], “unweighted” scoring approach [14, 15], and informally the “misery” score/index [16–18]. The term “level sum score” (LSS) was used in the recently published guidebook for analyzing EQ-5D data [16] and will be used for the remainder of this paper for consistency and clarity. The appeal of the LSS is its simplicity and consistency across populations (i.e., the same scoring system for all countries and populations).

Both the LSS and utility values are summary scores with similar limitations in interpretation; two patients may have the same summary score, but one may have extreme problems in a single dimension, whereas the other may have slight problems in several dimensions. Utility scores have found widespread acceptance over the LSS for the EQ-5D, potentially due to the rigorous development of preference elicitation.

The LSS has one major merit over utility scores when societal preference scores are unnecessary (i.e., non-economic applications): no algorithm is required to estimate the LSS, the end-user does not need to choose a specific value set to use (e.g., in multinational studies). Although previous investigations into the use of the EQ-5D LSS found substantial agreement and similar psychometric properties between the LSS and utility scores [13–15], the high correlations (ICC/Rho > 0.9) do not prove LSS accurately describes HRQoL or is appropriate for statistical inference. There is a dearth of literature specifically assessing the appropriateness of the LSS to describe HRQoL.

Item response theory (IRT) comprises a large set of models used to aid the construction and evaluation of multi-item scales. In general, these models assess the relationship between a latent variable of interest (θ) and the manifest/observable response patterns of a set of items. The probability of endorsing a particular response level on items of a scale is dependent on the respondent’s θ level. Parametric IRT has been previously applied to study the EQ-5D, although not to elucidate scoring [19–22]. Non-parametric item response theory (NP-IRT) approaches do not make strict assumptions about the shape of the function that describes the relationship between the response probability and the latent variable [23]. NP-IRT investigates whether the ordering of respondents along the summary score reflects the stochastic ordering of persons along θ [23, 24] instead of estimating θ. If the LSS is a proxy for θ (i.e., underlying health), then ordering of persons along the summary score is the ordering of persons along θ. Mokken scaling is a scaling approach comprising of a set of methods to assess whether the data fit a set of NP-IRT models. Two nested NP-IRT models included in Mokken scaling are as follows: the monotone homogeneity model (MHM), which examines ordering of persons along θ; and double monotonicity model (DMM), which examines ordering of persons and items along θ [25, 26]. If EQ-5D-5L data fit the MHM or DMM, then the use of LSS to represents underlying health can be justified and interpreted. The EQ-5D-5L is a good candidate for applying Mokken scaling as all items have the same number of ordered response categories with analogous adjectives.

The aims of these analyses were to investigate whether the MHM and DMM fit EQ-5D-5L data in order to 1) determine whether the LSS can be justified for the EQ-5D-5L and 2) examine whether an interpretation can be applied to such a score.

## Methods

### EQ-5D-5L

The EQ-5D health profile includes the items mobility (MO), self-care (SC), usual activities (UA), pain/discomfort (PD) and anxiety/depression (AD) [2]. The EQ-5D-5L asks respondents to endorse one of five response levels for each item: “no problems,” “slight problems,” “moderate problems,” “severe problems,” and “extreme problems”/ “unable to” [20, 27], describing 3125 (5^5^) health state profiles. The instrument also includes a visual analog scale (VAS) anchored by 0 (worst imaginable health) and 100 (best imaginable health) that is usually analyzed separately from the health profile.

The LSS is typically calculated by assigning a numerical value to each response level (i.e., 1 for “no problems”, 5 for “extreme problems”/”unable to”) and summing these values across the five items, resulting in a score from 5 (11,111, no problems on any dimension) to 25 (55,555, extreme problems on all dimensions) for the EQ-5D-5L.

#### Dataset

The Multi Instrument Comparison (MIC) project surveyed six countries in 2012 (Australia, Canada, Germany, Norway, UK, and USA), sampling respondents who self-reported seven chronic illnesses plus a healthy sample with no self-reported chronic conditions [28, 29]. Respondents completed a battery of health status, subjective well-being and capability measures, including the EQ-5D-5L. This dataset provides an opportunity to assess the scaling properties of the EQ-5D-5L in a large sample across disease and country subgroups. The disease groups chronic obstructive pulmonary disease and stroke were only sampled in the Australia and therefore excluded from analysis. All analyses were repeated by the subgroups self-reported disease and country.

Data management and descriptive statistics were handled in Microsoft Excel and Stata SE 13 [30], while all other analyses were conducted using the statistical language and environment R [31] with Van der Ark’s package “mokken” [32, 33]. The R script is included as supplementary material A. Permission to use the MIC dataset can be obtained here: https://www.aqol.com.au/index.php/mic-data.

#### Mokken scale analysis

We investigated the assumptions of two nested NP-IRT models that examine the ordinal location of patients and items along a single latent variable θ: respondents were ordered according to their LSS and items are ordered according to mean item scores [23, 25, 26]. The polytomous MHM and DMM models are extended from the dichotomous models [34, 35]. The MHM can elucidate whether a summary score can be used to order individuals along the latent variable. The more restrictive DMM is nested within the MHM and can further elucidate whether the items (i.e., EQ-5D-5L dimensions in these analyses) can be ordered invariantly along the latent variable. We examined how well polytomous MHM and DMM models fit EQ-5D-5L data.

#### Assessment of fit of the monotone homogeneity model

The MHM has three assumptions:

1. Unidimensionality: items within the scale measure the same underlying latent variable;
2. Local independence: responses to scale items are influenced only on level by θ; and
3. Monotonicity: probability of endorsing particular response levels is monotonically non-decreasing as θ increases.

Loevinger’s homogeneity coefficients, automated item selection procedure, and manifest monotonicity were used to assess the fit of the MHM to EQ-5D-5L data. Additionally, we examined scale reliability using Molenaar and Sijtsma’s rho (ρ) [36] and Guttman’s lamda-2 (λ-2) [37, 38].

Scalability of the EQ-5D-5L items was assessed using Loevinger’s scalability coefficients H, for which H values reflect item fit within a scale. H is measured on the item pair (H_ij_), item (H_i_), and scale (H_S_) levels. H_ij_ is the normed covariance between a pair of item scores while H_i_ is the normed covariance between item and rest scores [23, 32]. H_S_ is a weighted mean of H_i_. Negative H_ij_ and H_i_ coefficients indicate an item violates MHM. The closer H_i_ is to 1, the better an item can discriminate subjects along θ. On the item level, H_i_ > 0.3 is considered sufficient, while H_i_ > 5 indicates a strongly discriminating item. The commonly accepted rules of thumb for interpreting H_S_ were applied: H_S_ < 0.3 indicates the item set is unscalable, H_S_ between 0.3 and 0.4 indicates a weak scale, H_S_ between 0.4 and 0.5 indicates moderate, and H_S_ ≥ 0.5 indicates strong [25]. H_ij_ > 0 indicates that the data fit the MHM. We also used the H_ij_ to examine which item pairs are more strongly related than other pairs.

Automated item selection procedure (AISP) is a standard feature of the “mokken” package which selects subsets of items from a larger set that can represent attributes on which respondents can be ordered by total scores [32]. Although the lower bound of 0.3 is suggested for accepting items in a scale, it was more informative to determine at which level of H_i_ was items no longer scalable. Therefore, we first executed the AISP 12 times with the lower bound for H_i_ set between 0 and 0.5, increasing in steps of 0.05 [23, 32]. Then we pinpointed the level of H_i_ at which each of the five items was no longer appropriate for the scale by decreasing H_i_ in steps of 0.001 from the cutoff identified in the previous step.

#### Monotonicity

Latent monotonicity generally also implies manifest monotonicity, which is observable in the data [32] Therefore, if the LSS is a proxy for θ, then ordering of persons along the LSS reflects the ordering of persons along θ. Manifest monotonicity was assessed by examining whether the cumulative probability for a dimension-level rating at or above each dimension-level rating does not decrease across rest score groups. Rest scores are calculated by subtracting the item of interest from the LSS. Rest score groups are created automatically based on minimum sample size requirements for each group [32, 33]. Only violations greater than the default minimum (*minvi* = 0.03 for the function check.monotonicity of the R package “mokken”) were reported [32]. Furthermore, item step response functions (ISRFs) and item response functions (IRFs) were visually inspected for monotonicity. ISRF plots the probability for endorsing a response level or higher across the latent variable. IRF for polytomous items is the sum of an item’s ISRFs.

#### Assessment of invariant item ordering

The DMM model is a special case of MHM for which all assumptions of the MHM hold with an additional assumption that the IRF or ISRF of items does not intersect. Non-interception of ISRF is not necessarily evidence of item order [39] and would not be meaningful for interpretation of the LSS. Therefore, we did not examine non-interception of ISRF as a measure of DMM fit, rather focusing on invariant item ordering. Invariant item ordering can provide an interpretation: If the items have the same ordering along θ, then the summary score might be interpreted based on that order [32, 33, 39]. We therefore examined manifest invariant item ordering (MIIO) as suggested by Ligtvoet et al. (2010, 2011) [40, 41].

We assessed MIIO using the check.iio function of the R package “mokken,” which orders items by their conditional mean scores and checks each item pair for violations of ordering for rest score groups. Violations that exceed the default minimum value (number of ISRFs times 0.03) are reported [33, 41]. Coefficient H^T^ gives an indication of the degree to which the sample follows item ordering. We applied the rules of thumb that H^T^ < 0.3 implies the item ordering accuracy is too low, H^T^ between 0.3 and 0.4 as ordering with low accuracy, H^T^ between 0.4 and 0.5 as moderate accuracy, and H^T^ > 0.5 as highly accurate item ordering [41].

## Results

The included 7,933 subjects of the MIC reported 566 of the 3125 possible response patterns on the EQ-5D-5L; “11,111” (full health) and slight problems with PD with no problems on the other dimensions (“11,121”) were the first and second most often endorsed (19.3% and 14.3%, respectively). Subjects without chronic conditions were most homogeneous in regard to health profile (94 unique profiles), while those with diabetes reported the most diverse range of health (239 unique profiles; supplementary materials B and C). Number of distinct health profiles ranged from 164 (Norway) to 276 (UK) across country samples. Although over 8% of MIC respondents noted their general health as “poor,” endorsements of the most severe EQ-5D-5L levels were rare, especially for MO and SC (Table 1).

**Table 1:** Characteristics of the study sample (MIC)

Sample size	7933		Highest education			Health conditions		
	*n*	(%)		*n*	(%)		*n*	(%)
Female	4140	(52.19)	High school	2482	(31.29)	Healthy	1760	(22.19)
Age			Diploma/certificate/trade	3208	(40.44)	Asthma	856	(10.79)
18–24	513	(6.47)	University	2243	(28.27)	Cancer	772	(9.73)
25–34	943	(11.89)	Self-Rated Health			Depression	917	(11.56)
35–44	1133	(14.28)	Excellent	433	(5.46)	Diabetes	924	(11.65)
45–54	1672	(21.08)	Very Good	2089	(26.34)	Hearing problems	832	(10.49)
55–64	1977	(24.92)	Good	2726	(34.37)	Arthritis	929	(11.71)
65 +	1695	(21.37)	Fair	2039	(25.71)	Heart Conditions	943	(11.89)
			Poor	645	(8.13)			

EQ-5D-5L results										
	Mobility		Self-Care		Usual Activities		Pain/Discomfort		Anxiety Depression	
	*n*	(%)	*n*	(%)	*n*	(%)	*n*	(%)	*n*	(%)
No Problems/ None	5163	(65.08)	6984	(88.04)	5163	(65.08)	2331	(29.38)	3982	(50.20)
Slight (Problems)	1707	(21.52)	624	(7.87)	1707	(21.52)	3214	(40.51)	2319	(29.23)
Moderate (Problems)	771	(9.72)	258	(3.25)	771	(9.72)	1595	(20.11)	1088	(13.71)
Severe (Problems)	244	(3.08)	59	(0.74)	244	(3.08)	683	(8.61)	383	(4.83)
Unable to/ Extreme	48	(0.61)	8	(0.10)	48	(0.61)	110	(1.39)	161	(2.03)

### AISP and scalability

The EQ-5D-5L is a reliable scale, with ρ = 0.822 and λ-2 = 0.819. AISP placed all five items onto a single latent variable when the lower bound for H_i_ was set at the default 0.3, even when considering the 95% confidence interval (derived from standard errors). AD was identified as an unscalable item at H_i_ ≥ 0.378. PD was rejected from the scale at H_i_ ≥ 0.685, SC at H_i_ ≥ 0.721, and no items could be scaled at H_i_ ≥ 0.75 (Table 2).

**Table 2:** Item characteristics of the EQ-5D-5L

Item	Mean	H_i_	(SE)	Monotonicity			MIIO		
				AC	VI	Crit	AC	VI	Crit
1. Mobility	0.524	0.600	(0.008)	55	0	0	16	1	128
2. Self-Care	0.170	0.597	(0.010)	45	0	0	18	0	0
3. Usual Activities	0.526	0.647	(0.007)	40	0	0	16	1	150
4. Pain/Discomfort	1.121	0.603	(0.008)	36	0	0	13	0	0
5. Anxiety/Depression	0.793	0.377	(0.011)	40	0	0	15	2	216
H_s_		0.559	(0.007)						
Rho		0.822							
Lambda		0.819							
1. Mobility	0.524	0.731	(0.007)	36	0	0	9	0	0
2. Self-Care	0.170	0.681	(0.011)	33	0	0	10	0	0
3. Usual Activities	0.526	0.730	(0.007)	33	0	0	8	0	0
4. Pain/Discomfort	1.121	0.701	(0.008)	24	0	0	7	0	0
H_s_		0.714	(0.007)						
Rho		0.880							
Lambda		0.856							

*AC* Active Pairs, *VI* Violations, *Crit* Critical Values, *Hi* Coefficient H for items, *Hs* Coefficient H for the Scale, *MIIO* manifest invariant item ordering

H_i_ values were above 0.6 for all items except for AD, which had a H_i_ of 0.377. H_ij_ of AD with the other items ranged from 0.292 (MO) to 0.448 (UA) (Table 3). H_ij_ of SC and PD was larger than all AD item pairs, but smaller than 0.7, while all other item pairs had H_ij_ above 0.7. Because the H_i_ of AD was close to 0.3, the value of acceptability for H_i_, we decided to assess scalability by omitting this item. If the reduced item set would yield a much stronger scale, this would be an important finding. Researchers would possibly decide to employ the reduced items set in studies where a scale with increased scalability is needed, such as in instances where item ordering must be strictly maintained. When AD was removed from the model, H_S_ increased from 0.559 to 0.714, and the H_i_ of the four remaining items also increased (Table 2).

**Table 3:** Scalability coefficients and standard error for item pairs of the EQ-5D-5L

	Self-care		Usual activities		Pain/discomfort		Anxiety/depression	
	H_ij_	(SE)	H_ij_	(SE)	H_ij_	(SE)	H_ij_	(SE)
Mobility	0.705	(0.013)	0.750	(0.009)	0.725	(0.009)	0.292	(0.014)
Self-Care			0.718	(0.013)	0.617	(0.015)	0.364	(0.018)
Usual Activities					0.717	(0.009)	0.448	(0.013)
Pain/Discomfort							0.398	(0.013)

*H*_*ij*_ Coefficient H for item pairs, *SE* Standard Error

### Fit of the MHM model

**Fig. 1:**
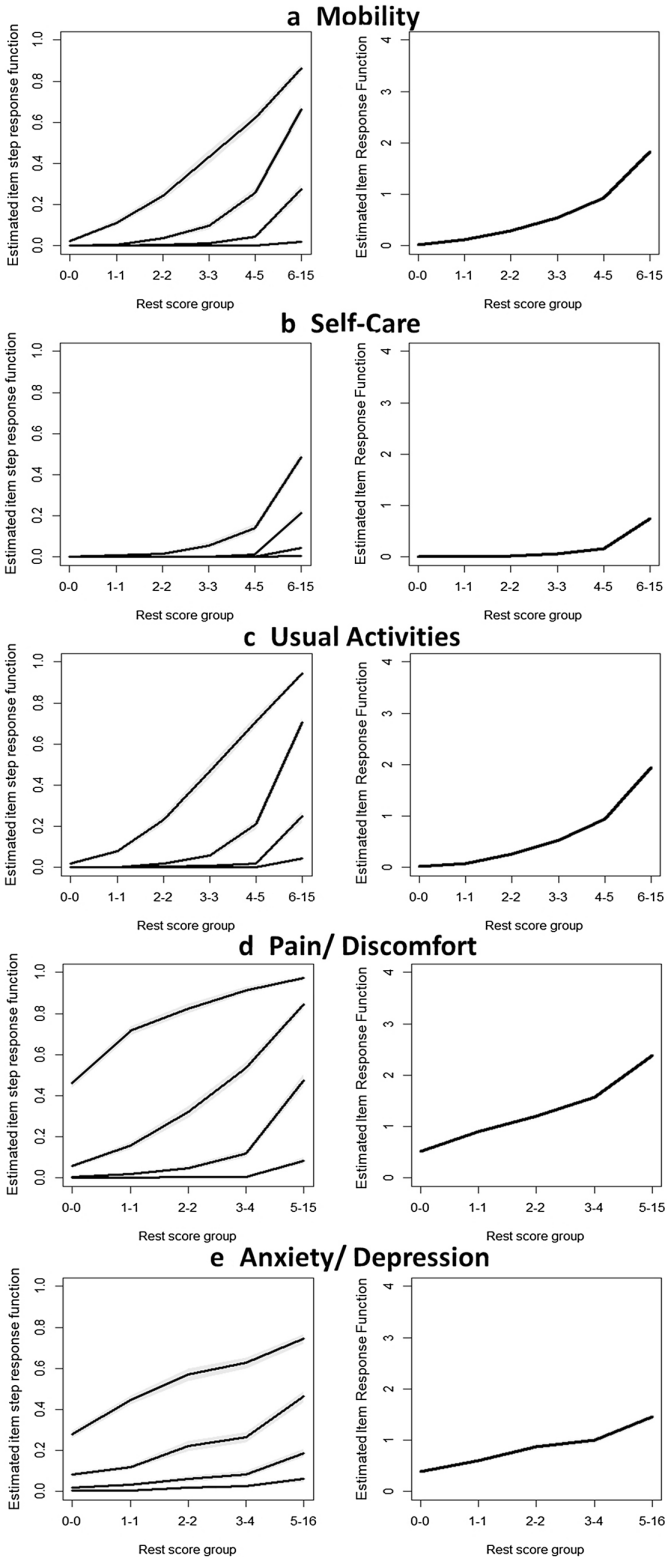
Item step response functions and item response functions of the five items of the EQ-5D-5L

Figure 1 illustrates the IRF and ISRF charted over rest score groups for the five items of the EQ-5D-5L. All IRFs and ISRFs increased monotonically with no violations of manifest monotonicity observed (Table 2). Critical values of all items were zero, showing no misfit of the MHM.

### Fit of MIIO

Two violations of MIIO were observed between 1) AD and MO, and 2) AD and UA (Table 4). AD had the highest critical value, and in backward selection was recommended for exclusion. Due to this recommendation for exclusion, we examined MIIO excluding the AD item, after which no violations of MIIO remained.

**Table 4:** EQ-5D-5L Item scaling coefficients stratified by disease type and country

	Mobility H_i_		Self-Care H_i_		Usual Activities H_i_		Pain/ Discomfort H_i_		Anxiety/ Depression H_i_	Scale H_S_		H^T^	
	Full scale	Without AD	Full Scale	Without AD	Full Scale	Without AD	Full Scale	Without AD	Full Scale	Full Scale	Without AD	Full Scale	Without AD
Complete Sample*	†0.600	0.731	0.597	0.681	†0.647	0.730	0.603	0.701	‡0.377	0.559	0.714	0.463	0.743
Healthy Sample	0.422	0.563	0.453	0.555	0.414	0.517	0.389	0.503	0.193	0.356	0.532	0.493	0.808
Self-Reported Chronic Condition													
Asthma*	†0.606	0.729	0.563	0.674	†0.635	0.731	0.601	0.687	‡0.355	0.549	0.709	0.470	0.760
Cancer*	†0.615	0.740	0.595	0.669	†0.642	0.734	0.598	0.683	‡0.366	0.561	0.711	0.467	0.712
Depression	0.426	0.592	0.484	0.565	0.511	0.592	0.408	0.584	0.230	0.393	0.585	0.747	0.651
Diabetes*	†0.627	0.747	0.610	0.700	†0.664	0.744	0.617	0.692	‡0.393	0.579	0.723	0.467	0.730
Hearing Problems*	†0.535	0.657	0.570	0.644	†0.568	0.667	0.524	0.594	‡0.305	0.492	0.640	0.534	0.797
Arthritis*	0.549	0.709	0.544	0.641	†0.599	0.713	0.559	0.660	†0.277	0.499	0.685	0.664	0.848
Heart Disease*	†0.628	0.745	0.636	0.721	†0.658	0.752	0.640	0.715	‡0.406	0.589	0.735	0.497	0.745
Country of survey sample													
Australia*	†0.582	0.752	0.570	0.653	†0.615	0.739	0.586	0.715	‡0.289	0.520	0.723	0.465	0.794
USA*	†0.602	0.715	0.595	0.667	†0.647	0.719	0.607	0.672	‡0.419	0.570	0.697	0.502	0.758
UK*	†0.663	0.805	0.650	0.758	†0.687	0.794	0.650	0.772	‡0.349	0.595	0.784	0.373	0.678
Canada	0.591	0.722	0.570	0.663	0.652	0.733	0.617	0.705	0.399	0.561	0.711	0.510	0.772
Norway	0.436	0.553	0.468	0.506	0.573	0.600	0.503	0.578	0.369	0.468	0.568	0.506	0.749
Germany*	†0.582	0.703	0.570	0.668	†0.615	0.699	0.586	0.675	‡0.289	0.520	0.688	0.467	0.732

*Backward item selection excluded AD; † one violation found; ‡ two violations found

H_i_: Coefficient H for items; H_s_: Coefficient H for the Scale; H^T^: Coefficient H for accuracy of item ordering

H^T^ calculated without exclusion due to backward item selection

**Fig. 2:**
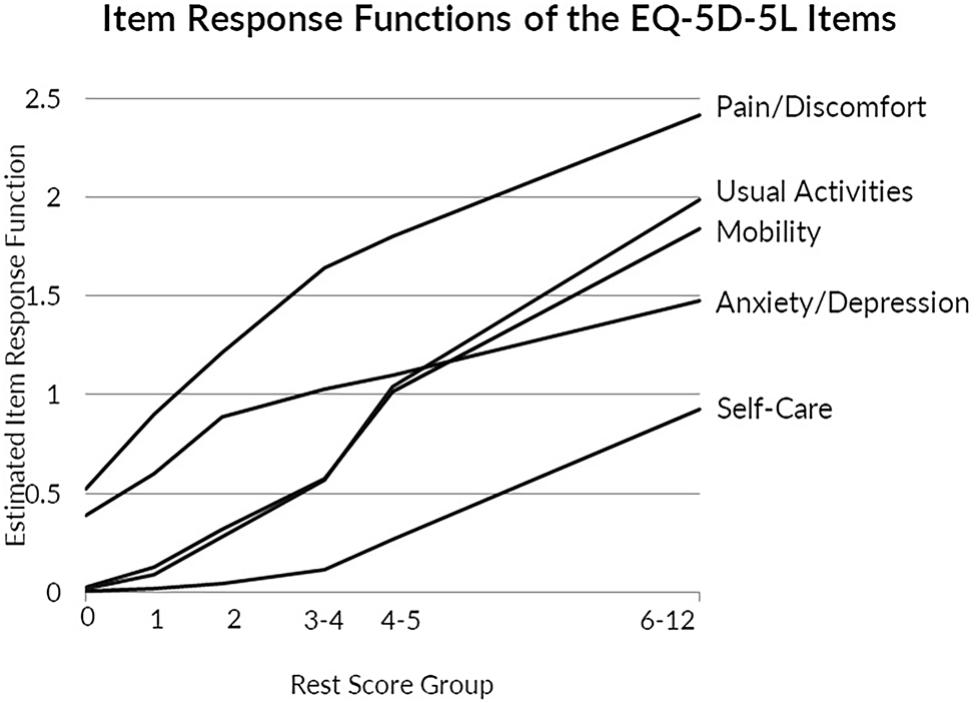
Item response functions of the five items of the EQ-5D-5L, estimated from paired restore groups

In order to visualize the IRF of all items in one figure, we selected item-pair results from the check.restscore function (Fig. 2). IRF charted over rest score groups indicate that the lower rest scores (≤ 3) were driven by PD and secondarily by AD. In the slightly higher rest score groups (2–4), the IRFs of MO and UA equally increased and overlapped, while AD’s IRF flattened. IRF of AD crossed both MO and US at rest scores 4–5. The IRF of SC did not increase until reaching higher rest score groups (4–5). Moderate item ordering was observed for the complete MIC sample (H^T^ = 0.463) (Table 4).

### Stratified analysis across subgroups

H coefficients were estimated for disease and country subgroups for the complete EQ-5D-5L scale as well as for the scale omitting the AD item as AD was recommended for exclusion by the check.iio procedure for many subgroups (Table 4). For the complete scale, H_s_ was weak for the healthy subsample (0.363), moderate for subjects with hearing problems and from Norway (0.496, 0.476, respectively). H_s_ for all other subgroups was “strong” but was all below 0.6.

**Fig. 3:**
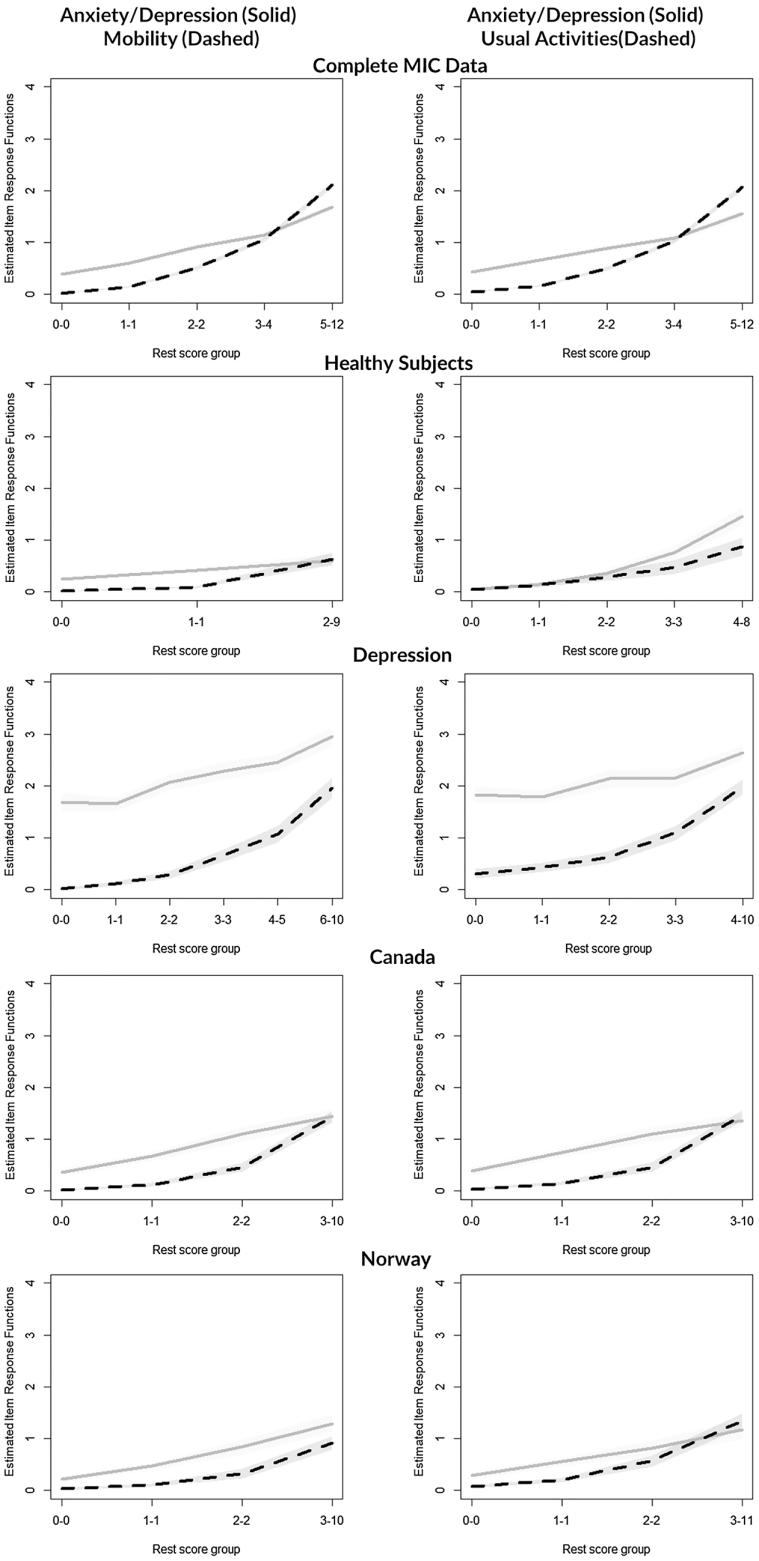
Paired item response functions of anxiety/depression with mobility and usual activities, across selected subgroups

H^T^ for the full scale ranged from 0.373 (Norway) to 0.747 (depression). Violations were found in the AD and MO and AD and UA pairs consistently across all subgroups except for respondents without self-reported chronic illness, depression, arthritis, and Canadian respondents (Table 4). Backward item selection recommended excluding AD for all subsamples that detected violations except for Norway. Critical values for Norway were 34 for UA and 50 for AD, demonstrating non-serious misfit. Figure 3 plots IRF of item pairs AD/MO and AD/UA for subgroups which did not recommend AD for removal. Not surprisingly, AD was easier to endorse at all rest score groups than MO or UA for the subgroup with depression, and the IRFs are far enough apart that they do not intersect.

H_ij_ tends to be largest between AD and UA, AD and PD across all subsamples except for healthy respondents, those reporting hearing problems and the Australian sample, showing that AD is more closely related to UA and PD than MO and SC (Table 5). H_ij_ between AD and all the other EQ-5D-5L items was particularly small for the healthy subsample.

**Table 5:** Item pair coefficient for anxiety/depression

	Mobility		Self-Care		Usual activities		Pain/Discomfort	
	H_ij_	(SE)	H_ij_	(SE)	H_ij_	(SE)	H_ij_	(SE)
Complete sample	0.292	(0.014)	0.364	(0.018)	0.448	(0.013)	0.398	(0.013)
Healthy sample	0.117	(0.043)	0.167	(0.080)	0.169	(0.047)	0.252	(0.034)
Self-reported chronic condition								
Asthma	0.297	(0.042)	0.249	(0.050)	0.395	(0.041)	0.419	(0.042)
Cancer	0.301	(0.042)	0.376	(0.053)	0.415	(0.040)	0.378	(0.042)
Depression	0.155	(0.023)	0.294	(0.034)	0.356	(0.029)	0.176	(0.023)
Diabetes	0.330	(0.036)	0.349	(0.052)	0.447	(0.037)	0.434	(0.034)
Hearing Problems	0.228	(0.048)	0.329	(0.072)	0.293	(0.051)	0.372	(0.047)
Arthritis	0.214	(0.029)	0.286	(0.037)	0.318	(0.030)	0.309	(0.030)
Heart Disease	0.355	(0.034)	0.386	(0.050)	0.423	(0.034)	0.455	(0.035)
Country of Survey Sample								
Australia	0.204	(0.033)	0.346	(0.048)	0.324	(0.035)	0.318	(0.033)
USA	0.334	(0.031)	0.390	(0.044)	0.470	(0.030)	0.467	(0.029)
UK	0.296	(0.031)	0.326	(0.040)	0.402	(0.032)	0.362	(0.030)
Canada	0.298	(0.034)	0.329	(0.048)	0.470	(0.032)	0.452	(0.030)
Norway	0.185	(0.038)	0.367	(0.056)	0.520	(0.036)	0.376	(0.034)
Germany	0.331	(0.031)	0.389	(0.036)	0.507	(0.028)	0.380	(0.031)

*H*_*ij*_ Coefficient H for item pairs, *SE* Standard Error

## Discussion

The EQ-5D-5L items form a strong Mokken scale, fitting the MHM and thus demonstrating that LSS, an additive summary score independent of population value sets, is acceptable and meaningful for measurement. These results empirically demonstrate that the EQ-5D-5L LSS orders respondents along a latent variable of health, with higher score indicating poorer health. The MHM fit of the EQ-5D-5L data reflects the rigorous work in questionnaire development, especially with refinement of the response levels [19, 27, 42]. Meijer and colleagues cautioned that sometimes strong Mokken scales are not optimal because they could reflect items covering similar or overlapping content [43, 44]. However, the EQ-5D is a brief scale with items covering diverse aspects of function and symptoms, so this concern is minimized.

MIIO results suggest that an interpretation of functional limitations and health symptoms can also be applied to the LSS: the low range of the score represents mainly problems with PD and AD, the lower to mid-range scores indicate additional problems with MO and UA, while the middle to higher scores reveal limitations in SC. The ordering of these items was found to be moderate. The finding that item ordering was not accurate for the healthy sub-sample reflected the observation of less variation in EQ-5D-5L responses in that subsample.

Our results empirically demonstrate what is conceptually understood: the LSS of the EQ-5D-5L orders persons by their levels of health. The relatively consistent performance of the EQ-5D-5L scale across countries is encouraging for the purpose of providing evidence to support the use of the LSS to compare the EQ-5D across countries. This is important because the EQ-5D has historically been scored using weights based on country-specific societal preferences. The LSS is used to describe data quality of valuation studies [45, 46] but has yet seen broader acceptance. A summary scoring function independent of population-specific value sets that is simple, psychometrically valid, and international in its applicability has tremendous advantages for researchers and population health scientists who wish to have a composite indicator of health for international comparisons using a measure available in hundreds of languages and is freely licensed and distributed by the EuroQol by non-profit organizations.

Although AD was initially retained in the scale as its H_i_ was above the commonly accepted cutoff of 0.3, it was excluded when the cutoff was only raised to above 0.378. Additionally, AD was found to violate MIIO in most subgroups—its IRF crosses the UA and MO IRFs at rest scores 3–4—and AD removal from the scale was suggested in backward model selection. The determination of whether an item should remain in a scale is not based solely on H_i_ but depends on conceptual and empirical considerations and the application of the instrument. When AD was omitted, H_s_ and H^T^ improved to above 0.7 to indicate very strong person and item ordering. Therefore, in applications where scalability or item ordering is required to be strong, one could apply the LSS to only the four physical items of the EQ-5D and assess the AD item separately. Although the EQ-5D is rarely used as a diagnostic tool on the level of individual patients, item ordering can still be relevant for group level applications. For example, although patient groups with mainly physical symptoms do not suffer from anxiety/depressive problems more than the general population, the AD item may be more difficult to endorse than the physical items at moderate or more severe levels of disease (as indicated in these results). However, for conditions for which mental health is affected, the AD item could be easier to endorse than MO, SC and UA across the scale (as supported by our findings of MIIO in the subgroup with depression). The relationship between items may also be modified by other factors such as age or gender. This is an area needing future research.

IRT approaches to evaluating the EQ-5D have been relatively scarce in the literature: our results are comparable to available evidence. A recent investigation of the EQ-5D using Rasch rating scale model reported similar item ordering as our findings: PD was the easiest to endorse, UA, AD, and MO are at middle levels of difficulty of endorsement, and SC was the most difficult to endorse item [21]. Our scalability results were similar to previously published results for the physical function subscale of the SF-36—H_S_ of 0.69 and H^T^ of 0.53 [44].

IRT assumes items are indicators of a single latent variable. However, the EQ-5D was constructed using five different dimensions to create a composite measure of health status. AD conceptually measures mental health, while the other four items address physical health [48–50]. A previous study revealed that when several health measures were modeled with the EQ-5D-5L, MO, SC, and UA belonged to one dimension, AD to a second, and PD to a third [51]. However, other investigations found sufficient evidence that self-reported physical and mental health can be summarized using a single score [52]. Recent confirmatory factor analysis found the model including all five EQ-5D-5L items to have acceptable fit statistics [47]. These previous findings along with this study illustrate the tension between the multidimensional nature of health and summarizing health as a single latent construct. The theoretical measurement model, such as whether the EQ-5D is a formative or reflective measurement [47, 54, 55], must be considered when applying scoring approaches.

A limitation of this study was that the dataset only included adult participants from Western, developed countries. If person and item ordering are dependent on how item descriptions and response categories are interpreted, then these results may not extend to other populations. Further, the data were collected via online survey panels, and such participants may differ from the general population [29]. There is also a pressing need to conduct similar research in children. Due to ethical, methodological, and conceptual problems involved in eliciting preferences for children, the version of the EQ-5D for children and adolescents (EQ-5D-Y) does not have a preference value set [53]. Therefore, application of the LSS may be particularly relevant for the EQ-5D-Y as its use expands.

## Conclusion

A conceptually cohesive scale of health can be operationalized using the LSS using all five items of the EQ-5D-5L as higher LSS scores indicate worse health and more severe functional limitations. In general, lower range of the score represents mainly problems with pain, the mid-range indicates additional problems with mobility and usual activities, and middle to higher range of scores reveals additional limitations with self-care. Anxiety/depression is easier to endorse than MO or UA at the lower range of scores, but at moderate and higher scores becomes more difficult to endorse. Compared to utility scores, LSS scores have advantages depending on the application and subgroup/population. However, the scale is weak in the healthy subsample, indicating it may be less informative in such populations. More work must be done to investigate whether person and item order holds for other populations, especially for children and adolescents.

## Supplementary Information

Below is the link to the electronic supplementary material.

Supplementary file1 (R 12 KB)

Supplementary file2 (DOCX 48 KB)

Supplementary file3 (DOCX 24 KB)
