# Supplementary material for: Combining EQ-5D-5L items into a level summary score: demonstrating feasibility using non-parametric item response theory using an international dataset
Source: Qual Life Res. 2021 Jul 8;31(1):11–23. doi: 10.1007/s11136-021-02922-1 (PMC8800896; doi:10.1007/s11136-021-02922-1)
Supplement: Supplementary file 2 — Supplementary file2 (DOCX 48 KB) [file 11136_2021_2922_MOESM2_ESM.docx]

| Supplementary Material Table 1B: EQ-5D-5L Characteristics Across Health Conditions of the MIC Dataset | | | | | | | | | | | | | | | | | | |
| --- | --- | --- | --- | --- | --- | --- | --- | --- | --- | --- | --- | --- | --- | --- | --- | --- | --- | --- |
|  | Total Sample | | Healthy Sample | | Asthma | | Cancer | | Depression | | Diabetes | | Hearing Problems | | Arthritis | | Heart Disease | |
|  | n | (%) | n | (%) | n | (%) | n | (%) | n | (%) | n | (%) | n | (%) | n | (%) | n | (%) |
| Total | 7,933 |  | 1,760 |  | 856 |  | 772 |  | 917 |  | 924 |  | 832 |  | 929 |  | 943 |  |
| Self Rated Health |  |  |  |  |  |  |  |  |  |  |  |  |  |  |  |  |  |  |
| Excellent | 433 | (5.5) | 278 | (15.8) | 26 | (3.0) | 20 | (2.6) | 14 | (1.5) | 7 | (0.8) | 59 | (7.1) | 16 | (1.7) | 13 | (1.4) |
| Very Good | 2,089 | (26.3) | 866 | (49.2) | 219 | (25.6) | 127 | (16.5) | 103 | (11.2) | 163 | (17.6) | 280 | (33.7) | 162 | (17.4) | 169 | (17.9) |
| Good | 2,726 | (34.4) | 533 | (30.3) | 331 | (38.7) | 250 | (32.4) | 323 | (35.2) | 310 | (33.5) | 318 | (38.2) | 347 | (37.4) | 314 | (33.3) |
| Fair | 2,039 | (25.7) | 82 | (4.7) | 211 | (24.6) | 283 | (36.7) | 339 | (37.0) | 333 | (36.0) | 152 | (18.3) | 308 | (33.2) | 331 | (35.1) |
| Poor | 645 | (8.1) | 1 | (0.1) | 69 | (8.1) | 92 | (11.9) | 138 | (15.0) | 111 | (12.0) | 22 | (2.6) | 96 | (10.3) | 116 | (12.3) |
| Mobility |  |  |  |  |  |  |  |  |  |  |  |  |  |  |  |  |  |  |
| None | 5,163 | (65.1) | 1,605 | (91.2) | 604 | (70.6) | 440 | (57.0) | 593 | (64.7) | 531 | (57.5) | 628 | (75.5) | 369 | (39.7) | 544 | (57.7) |
| Slight | 1,707 | (21.5) | 123 | (7.0) | 156 | (18.2) | 167 | (21.6) | 187 | (20.4) | 196 | (21.2) | 138 | (16.6) | 301 | (32.4) | 199 | (21.1) |
| Moderate | 771 | (9.7) | 22 | (1.3) | 69 | (8.1) | 116 | (15.0) | 103 | (11.2) | 134 | (14.5) | 46 | (5.5) | 170 | (18.3) | 136 | (14.4) |
| Severe | 244 | (3.1) | 8 | (0.5) | 24 | (2.8) | 45 | (5.8) | 32 | (3.5) | 57 | (6.2) | 17 | (2.0) | 83 | (8.9) | 62 | (6.6) |
| unable to | 48 | (0.6) | 2 | (0.1) | 3 | (0.4) | 4 | (0.5) | 2 | (0.2) | 6 | (0.6) | 3 | (0.4) | 6 | (0.6) | 2 | (0.2) |
| Self-Care |  |  |  |  |  |  |  |  |  |  |  |  |  |  |  |  |  |  |
| None | 6,984 | (88.0) | 1,727 | (98.1) | 753 | (88.0) | 662 | (85.8) | 756 | (82.4) | 778 | (84.2) | 781 | (93.9) | 713 | (76.7) | 814 | (86.3) |
| Slight | 624 | (7.9) | 23 | (1.3) | 74 | (8.6) | 62 | (8.0) | 104 | (11.3) | 91 | (9.8) | 36 | (4.3) | 152 | (16.4) | 82 | (8.7) |
| Moderate | 258 | (3.3) | 4 | (0.2) | 25 | (2.9) | 40 | (5.2) | 42 | (4.6) | 41 | (4.4) | 11 | (1.3) | 54 | (5.8) | 41 | (4.3) |
| Severe | 59 | (0.7) | 4 | (0.2) | 4 | (0.5) | 6 | (0.8) | 14 | (1.5) | 12 | (1.3) | 4 | (0.5) | 9 | (1.0) | 6 | (0.6) |
| unable to | 8 | (0.1) | 2 | (0.1) | 0 | (0.0) | 2 | (0.3) | 1 | (0.1) | 2 | (0.2) | 0 | (0.0) | 1 | (0.1) | 0 | (0.0) |
| Usual Activities |  |  |  |  |  |  |  |  |  |  |  |  |  |  |  |  |  |  |
| None | 5,163 | (65.1) | 1,619 | (92.0) | 582 | (68.0) | 409 | (53.0) | 433 | (47.2) | 562 | (60.8) | 638 | (76.7) | 384 | (41.3) | 536 | (56.8) |
| Slight | 1,707 | (21.5) | 122 | (6.9) | 178 | (20.8) | 201 | (26.0) | 270 | (29.4) | 200 | (21.6) | 144 | (17.3) | 350 | (37.7) | 242 | (25.7) |
| Moderate | 771 | (9.7) | 15 | (0.9) | 73 | (8.5) | 116 | (15.0) | 146 | (15.9) | 121 | (13.1) | 39 | (4.7) | 142 | (15.3) | 119 | (12.6) |
| Severe | 244 | (3.1) | 3 | (0.2) | 20 | (2.3) | 39 | (5.1) | 55 | (6.0) | 37 | (4.0) | 9 | (1.1) | 39 | (4.2) | 42 | (4.5) |
| unable to | 48 | (0.6) | 1 | (0.1) | 3 | (0.4) | 7 | (0.9) | 13 | (1.4) | 4 | (0.4) | 2 | (0.2) | 14 | (1.5) | 4 | (0.4) |
| Pain/Discomfort |  |  |  |  |  |  |  |  |  |  |  |  |  |  |  |  |  |  |
| None | 2,331 | (29.4) | 967 | (54.9) | 269 | (31.4) | 160 | (20.7) | 204 | (22.2) | 235 | (25.4) | 254 | (30.5) | 34 | (3.7) | 208 | (22.1) |
| Slight | 3,214 | (40.5) | 679 | (38.6) | 363 | (42.4) | 337 | (43.7) | 340 | (37.1) | 346 | (37.4) | 402 | (48.3) | 350 | (37.7) | 397 | (42.1) |
| Moderate | 1,595 | (20.1) | 95 | (5.4) | 158 | (18.5) | 177 | (22.9) | 244 | (26.6) | 219 | (23.7) | 131 | (15.7) | 348 | (37.5) | 223 | (23.6) |
| Severe | 683 | (8.6) | 19 | (1.1) | 61 | (7.1) | 83 | (10.8) | 106 | (11.6) | 99 | (10.7) | 40 | (4.8) | 176 | (18.9) | 99 | (10.5) |
| Extreme | 110 | (1.4) | 0 | (0.0) | 5 | (0.6) | 15 | (1.9) | 23 | (2.5) | 25 | (2.7) | 5 | (0.6) | 21 | (2.3) | 16 | (1.7) |
| Anxiety Depression | |  |  |  |  |  |  |  |  |  |  |  |  |  |  |  |  |  |
| Not | 3,982 | (50.2) | 1,246 | (70.8) | 426 | (49.8) | 366 | (47.4) | 45 | (4.9) | 450 | (48.7) | 483 | (58.1) | 477 | (51.3) | 489 | (51.9) |
| Slight | 2,319 | (29.2) | 427 | (24.3) | 293 | (34.2) | 253 | (32.8) | 234 | (25.5) | 273 | (29.5) | 259 | (31.1) | 300 | (32.3) | 280 | (29.7) |
| Moderate | 1,088 | (13.7) | 71 | (4.0) | 95 | (11.1) | 110 | (14.2) | 338 | (36.9) | 146 | (15.8) | 76 | (9.1) | 121 | (13.0) | 131 | (13.9) |
| Severe | 383 | (4.8) | 13 | (0.7) | 29 | (3.4) | 35 | (4.5) | 201 | (21.9) | 38 | (4.1) | 11 | (1.3) | 26 | (2.8) | 30 | (3.2) |
| Extreme | 161 | (2.0) | 3 | (0.2) | 13 | (1.5) | 8 | (1.0) | 99 | (10.8) | 17 | (1.8) | 3 | (0.4) | 5 | (0.5) | 13 | (1.4) |
|  |  |  |  |  |  |  |  |  |  |  |  |  |  |  |  |  |  |  |
| Number of Reported Health Profiles | 566 |  | 94 |  | 159 |  | 195 |  | 239 |  | 207 |  | 118 |  | 206 |  | 199 |  |
| Most frequently reported profile | 11111 | | 11111 | | 11111 | | 11121 | | 11122 | | 11111 | | 11111 | | 11121 | | 11121 | |
|  | 1,530 | (19.3) | 738 | (41.9) | 178 | (20.8) | 105 | (13.6) | 75 | (8.2) | 161 | (17.4) | 180 | (21.6) | 138 | (14.9) | 147 | (15.6) |
| 2nd most frequently reported profile | 11121 | | 11121 | | 11121 | | 11111 | | 11113 | | 11121 | | 11121 | | 21232 | | 11111 | |
|  | 1,135 | (14.3) | 346 | (19.7) | 118 | (13.8) | 92 | (11.9) | 57 | (6.2) | 102 | (11.0) | 165 | (19.8) | 39 | (4.2) | 137 | (14.5) |
| 3rd most frequently reported profile | 11122 | | 11122 | | 11122 | | 11122 | | 11123 | | 11122 | | 11122 | | 11122 | | 11122 | |
|  | 672 | (8.5) | 174 | (9.9) | 96 | (11.2) | 60 | (7.8) | 50 | (5.5) | 72 | (7.8) | 94 | (11.3) | 37 | (4.0) | 64 | (6.8) |
| Full Health (11111) | 1,530 | (19.3) | 738 | (41.9) | 178 | (20.8) | 92 | (11.9) | 18 | (2.0) | 161 | (17.4) | 180 | (21.6) | 26 | (2.8) | 137 | (14.5) |

| Supplementary Material Table 1C: EQ-5D-5L Characteristics Across Country Subsamples of the MIC Dataset | | | | | | | | | | | | | | |
| --- | --- | --- | --- | --- | --- | --- | --- | --- | --- | --- | --- | --- | --- | --- |
|  | Total Sample | | Australia | | USA | | UK | | Canada | | Norway | | Germany | |
|  | n | (%) | n | (%) | n | (%) | n | (%) | n | (%) | n | (%) | n | (%) |
| Total | 7,933 |  | 1,341 |  | 1,460 |  | 1,356 |  | 1,330 |  | 1,177 |  | 1,269 |  |
| Self Rated Health |  |  |  |  |  |  |  |  |  |  |  |  |  |  |
| Excellent | 433 | (5.5) | 57 | (4.3) | 92 | (6.3) | 62 | (4.6) | 86 | (6.5) | 100 | (8.5) | 36 | (2.8) |
| Very Good | 2,089 | (26.3) | 361 | (26.9) | 415 | (28.4) | 334 | (24.6) | 407 | (30.6) | 342 | (29.1) | 230 | (18.1) |
| Good | 2,726 | (34.4) | 449 | (33.5) | 490 | (33.6) | 403 | (29.7) | 463 | (34.8) | 392 | (33.3) | 529 | (41.7) |
| Fair | 2,039 | (25.7) | 364 | (27.1) | 380 | (26.0) | 372 | (27.4) | 297 | (22.3) | 229 | (19.5) | 397 | (31.3) |
| Poor | 645 | (8.1) | 110 | (8.2) | 83 | (5.7) | 185 | (13.6) | 77 | (5.8) | 113 | (9.6) | 77 | (6.1) |
| Mobility |  |  |  |  |  |  |  |  |  |  |  |  |  |  |
| None | 5,163 | (65.1) | 858 | (64.0) | 927 | (63.5) | 829 | (61.1) | 932 | (70.1) | 952 | (80.9) | 816 | (64.3) |
| Slight | 1,707 | (21.5) | 275 | (20.5) | 298 | (20.4) | 251 | (18.5) | 225 | (16.9) | 155 | (13.2) | 263 | (20.7) |
| Moderate | 771 | (9.7) | 149 | (11.1) | 167 | (11.4) | 159 | (11.7) | 133 | (10.0) | 54 | (4.6) | 134 | (10.6) |
| Severe | 244 | (3.1) | 53 | (4.0) | 59 | (4.0) | 112 | (8.3) | 35 | (2.6) | 14 | (1.2) | 55 | (4.3) |
| unable to | 48 | (0.6) | 6 | (0.4) | 9 | (0.6) | 5 | (0.4) | 5 | (0.4) | 2 | (0.2) | 1 | (0.1) |
| Self-Care |  |  |  |  |  |  |  |  |  |  |  |  |  |  |
| None | 6,984 | (88.0) | 1,199 | (89.4) | 1,284 | (87.9) | 1,135 | (83.7) | 1,195 | (89.8) | 1,103 | (93.7) | 1,068 | (84.2) |
| Slight | 624 | (7.9) | 97 | (7.2) | 114 | (7.8) | 126 | (9.3) | 97 | (7.3) | 61 | (5.2) | 129 | (10.2) |
| Moderate | 258 | (3.3) | 36 | (2.7) | 47 | (3.2) | 72 | (5.3) | 27 | (2.0) | 11 | (0.9) | 65 | (5.1) |
| Severe | 59 | (0.7) | 9 | (0.7) | 14 | (1.0) | 18 | (1.3) | 9 | (0.7) | 2 | (0.2) | 7 | (0.6) |
| unable to | 8 | (0.1) | 0 | (0.0) | 1 | (0.1) | 5 | (0.4) | 2 | (0.2) | 0 | (0.0) | 0 | (0.0) |
| Usual Activities |  |  |  |  |  |  |  |  |  |  |  |  |  |  |
| None | 5,163 | (65.1) | 865 | (64.5) | 923 | (63.2) | 852 | (62.8) | 903 | (67.9) | 844 | (71.7) | 776 | (61.2) |
| Slight | 1,707 | (21.5) | 306 | (22.8) | 337 | (23.1) | 270 | (19.9) | 255 | (19.2) | 242 | (20.6) | 297 | (23.4) |
| Moderate | 771 | (9.7) | 129 | (9.6) | 150 | (10.3) | 145 | (10.7) | 138 | (10.4) | 66 | (5.6) | 143 | (11.3) |
| Severe | 244 | (3.1) | 35 | (2.6) | 38 | (2.6) | 70 | (5.2) | 30 | (2.3) | 22 | (1.9) | 49 | (3.9) |
| unable to | 48 | (0.6) | 6 | (0.4) | 12 | (0.8) | 19 | (1.4) | 4 | (0.3) | 3 | (0.3) | 4 | (0.3) |
| Pain/Discomfort |  |  |  |  |  |  |  |  |  |  |  |  |  |  |
| None | 2,331 | (29.4) | 374 | (27.9) | 380 | (26.0) | 434 | (32.0) | 415 | (31.2) | 400 | (34.0) | 328 | (25.8) |
| Slight | 3,214 | (40.5) | 554 | (41.3) | 569 | (39.0) | 504 | (37.2) | 519 | (39.0) | 527 | (44.8) | 541 | (42.6) |
| Moderate | 1,595 | (20.1) | 281 | (21.0) | 358 | (24.5) | 254 | (18.7) | 274 | (20.6) | 176 | (15.0) | 252 | (19.9) |
| Severe | 683 | (8.6) | 114 | (8.5) | 128 | (8.8) | 132 | (9.7) | 111 | (8.3) | 67 | (5.7) | 131 | (10.3) |
| Extreme | 110 | (1.4) | 18 | (1.3) | 25 | (1.7) | 32 | (2.4) | 11 | (0.8) | 7 | (0.6) | 17 | (1.3) |
| Anxiety Depression | |  |  |  |  |  |  |  |  |  |  |  |  |  |
| Not | 3,982 | (50.2) | 685 | (51.1) | 676 | (46.3) | 594 | (43.8) | 627 | (47.1) | 721 | (61.3) | 679 | (53.5) |
| Slight | 2,319 | (29.2) | 383 | (28.6) | 460 | (31.5) | 434 | (32.0) | 428 | (32.2) | 280 | (23.8) | 334 | (26.3) |
| Moderate | 1,088 | (13.7) | 191 | (14.2) | 229 | (15.7) | 217 | (16.0) | 177 | (13.3) | 114 | (9.7) | 160 | (12.6) |
| Severe | 383 | (4.8) | 52 | (3.9) | 62 | (4.2) | 78 | (5.8) | 63 | (4.7) | 50 | (4.2) | 78 | (6.1) |
| Extreme | 161 | (2.0) | 30 | (2.2) | 33 | (2.3) | 33 | (2.4) | 35 | (2.6) | 12 | (1.0) | 18 | (1.4) |
|  |  |  |  |  |  |  |  |  |  |  |  |  |  |  |
| Number of Reported Health Profiles | 566 |  | 212 |  | 249 |  | 276 |  | 222 |  | 164 |  | 239 |  |
| Most frequently reported profile | 11111 | | 11111 | | 11111 | | 11111 | | 11111 | | 11111 | | 11111 | |
|  | 1,530 | (19.3) | 241 | (18.0) | 251 | (17.2) | 219 | (16.2) | 288 | (21.7) | 297 | (25.2) | 234 | (18.4) |
| 2nd most frequently reported profile | 11121 | | 11121 | | 11121 | | 11121 | | 11121 | | 11121 | | 11121 | |
|  | 1,135 | (14.3) | 181 | (13.5) | 191 | (13.1) | 150 | (11.1) | 168 | (12.6) | 230 | (19.5) | 215 | (16.9) |
| 3rd most frequently reported profile | 11122 | | 11121 | | 11121 | | 11121 | | 11121 | | 11121 | | 11121 | |
|  | 672 | (8.5) | 181 | (13.5) | 191 | (13.1) | 150 | (11.1) | 168 | (12.6) | 230 | (19.5) | 215 | (16.9) |
| Full Health (11111) | 1,530 | (19.3) | 241 | (18.0) | 251 | (17.2) | 219 | (16.2) | 288 | (21.7) | 297 | (25.2) | 234 | (18.4) |
