# Supplementary material for: Combining EQ-5D-5L items into a level summary score: demonstrating feasibility using non-parametric item response theory using an international dataset
Source: Qual Life Res. 2021 Jul 8;31(1):11–23. doi: 10.1007/s11136-021-02922-1 (PMC8800896; doi:10.1007/s11136-021-02922-1)
Supplement: Supplementary file 3 — Supplementary file3 (DOCX 24 KB) [file 11136_2021_2922_MOESM3_ESM.docx]

Supplementary Material D: Table 4S: EQ-5D-5L Item Scaling Coefficients with Standard Errors Stratified by Disease Type and Country

| Table 4S: EQ-5D-5L Item Scaling Coefficients with Standard Errors Stratified by Disease Type and Country: Coefficients (SE) | | | | | | | | | | | | | | | | | | | | | | | | | |
| --- | --- | --- | --- | --- | --- | --- | --- | --- | --- | --- | --- | --- | --- | --- | --- | --- | --- | --- | --- | --- | --- | --- | --- | --- | --- |
|  | Mobility H_i_ | | | | Self-Care H_i_ | | | | Usual Activities H_i_ | | | | Pain/ Discomfort H_i_ | | | | | Anxiety/ Depression H_i_ | | Scale H_S_ | | | | H^T^ | |
|  | Full Scale | | Without AD | | Full Scale | | Without AD | | Full Scale | | Without AD |  | Full Scale | | | Without AD | | Full Scale | | Full Scale | | Without AD | | Full Scale | Without AD |
| Complete Sample* | †0.600 | (0.008) | 0.731 | (0.014) | 0.597 | (0.010) | 0.681 | (0.018) | †0.647 | (0.007) | 0.730 | (0.013) | 0.603 | (0.008) | 0.701 | | (0.013) | ‡0.377 | (0.011) | 0.559 | (0.007) | 0.714 | (0.007) | 0.463 | 0.743 |
| Healthy Sample | 0.422 | (0.036) | 0.563 | (0.043) | 0.453 | (0.068) | 0.555 | (0.080) | 0.414 | (0.041) | 0.517 | (0.047) | 0.389 | (0.032) | 0.503 | | (0.034) | 0.193 | (0.035) | 0.356 | (0.034) | 0.532 | (0.040) | 0.493 | 0.808 |
| Self-Reported Chronic Condition | | | | | | | | | | | | | | | | | | | | | | | | | |
| Asthma* | †0.606 | (0.024) | 0.729 | (0.042) | 0.563 | (0.028) | 0.674 | (0.050) | †0.635 | (0.021) | 0.731 | (0.041) | 0.601 | (0.024) | 0.687 | | (0.042) | ‡0.355 | (0.035) | 0.549 | (0.023) | 0.709 | (0.022) | 0.470 | 0.760 |
| Cancer* | †0.615 | (0.020) | 0.740 | (0.042) | 0.595 | (0.030) | 0.669 | (0.053) | †0.642 | (0.021) | 0.734 | (0.040) | 0.598 | (0.023) | 0.683 | | (0.042) | ‡0.366 | (0.036) | 0.561 | (0.022) | 0.711 | (0.019) | 0.467 | 0.712 |
| Depression | 0.426 | (0.023) | 0.592 | (0.023) | 0.484 | (0.026) | 0.565 | (0.034) | 0.511 | (0.020) | 0.592 | (0.029) | 0.408 | (0.022) | 0.584 | | (0.023) | 0.230 | (0.021) | 0.393 | (0.020) | 0.585 | (0.022) | 0.747 | 0.651 |
| Diabetes* | †0.627 | (0.020) | 0.747 | (0.036) | 0.610 | (0.028) | 0.700 | (0.052) | †0.664 | (0.017) | 0.744 | (0.037) | 0.617 | (0.020) | 0.692 | | (0.034) | ‡0.393 | (0.032) | 0.579 | (0.019) | 0.723 | (0.019) | 0.467 | 0.730 |
| Hearing Problems* | †0.535 | (0.030) | 0.657 | (0.048) | 0.570 | (0.041) | 0.644 | (0.072) | †0.568 | (0.029) | 0.667 | (0.051) | 0.524 | (0.032) | 0.594 | | (0.047) | ‡0.305 | (0.043) | 0.492 | (0.030) | 0.640 | (0.031) | 0.534 | 0.797 |
| Arthritis* | 0.549 | (0.018) | 0.709 | (0.029) | 0.544 | (0.022) | 0.641 | (0.037) | †0.599 | (0.015) | 0.713 | (0.030) | 0.559 | (0.019) | 0.660 | | (0.030) | †0.277 | (0.026) | 0.499 | (0.017) | 0.685 | (0.017) | 0.664 | 0.848 |
| Heart Disease* | †0.628 | (0.019) | 0.745 | (0.034) | 0.636 | (0.026) | 0.721 | (0.050) | †0.658 | (0.018) | 0.752 | (0.034) | 0.640 | (0.020) | 0.715 | | (0.035) | ‡0.406 | (0.031) | 0.589 | (0.019) | 0.735 | (0.018) | 0.497 | 0.745 |
| Country of Survey Sample | | | | | | | | | | | | | | | | | | | | | | | | | |
| Australia* | †0.582 | (0.018) | 0.752 | (0.033) | 0.570 | (0.026) | 0.653 | (0.048) | †0.615 | (0.017) | 0.739 | (0.035) | 0.586 | (0.019) | 0.715 | | (0.033) | ‡0.289 | (0.030) | 0.520 | (0.019) | 0.723 | (0.016) | 0.465 | 0.794 |
| USA* | †0.602 | (0.018) | 0.715 | (0.031) | 0.595 | (0.023) | 0.667 | (0.044) | †0.647 | (0.017) | 0.719 | (0.030) | 0.607 | (0.018) | 0.672 | | (0.029) | ‡0.419 | (0.026) | 0.570 | (0.017) | 0.697 | (0.016) | 0.502 | 0.758 |
| UK* | †0.663 | (0.015) | 0.805 | (0.031) | 0.650 | (0.022) | 0.758 | (0.040) | †0.687 | (0.013) | 0.794 | (0.032) | 0.650 | (0.016) | 0.772 | | (0.030) | ‡0.349 | (0.028) | 0.595 | (0.016) | 0.784 | (0.014) | 0.373 | 0.678 |
| Canada | 0.591 | (0.020) | 0.722 | (0.034) | 0.570 | (0.032) | 0.663 | (0.048) | 0.652 | (0.017) | 0.733 | (0.032) | 0.617 | (0.019) | 0.705 | | (0.030) | 0.399 | (0.028) | 0.561 | (0.019) | 0.711 | (0.018) | 0.510 | 0.772 |
| Norway | 0.436 | (0.027) | 0.553 | (0.038) | 0.468 | (0.033) | 0.506 | (0.056) | 0.573 | (0.018) | 0.600 | (0.036) | 0.503 | (0.024) | 0.578 | | (0.034) | 0.369 | (0.027) | 0.468 | (0.019) | 0.568 | (0.026) | 0.506 | 0.749 |
| Germany* | †0.582 | (0.018) | 0.703 | (0.031) | 0.570 | (0.019) | 0.668 | (0.036) | †0.615 | (0.016) | 0.699 | (0.028) | 0.586 | (0.019) | 0.675 | | (0.031) | ‡0.289 | (0.025) | 0.520 | (0.017) | 0.688 | (0.016) | 0.467 | 0.732 |
| * Backward item selection excluded AD; † one violation found; ‡ two violations found  H_i_: Coefficient H for items; H_s_: Coefficient H for the Scale; H^T^: Coefficient H for accuracy of item ordering; SE: Standard Error  H^T^ calculated without exclusion due to backward item selection | | | | | | | | | | | | | | | | | | | | | | | | | |
